# Extent of Surgery and Implications of Transection Margin Status after Resection of IPMNs

**DOI:** 10.1155/2014/269803

**Published:** 2014-09-04

**Authors:** Marina Paini, Stefano Crippa, Filippo Scopelliti, Andrea Baldoni, Alberto Manzoni, Giulio Belfiori, Stefano Partelli, Massimo Falconi

**Affiliations:** ^1^Division of Pancreatic Surgery, Ospedali Riuniti, Università Politecnica delle Marche, Via Conca 71, 60126 Ancona, Italy; ^2^Division of Pancreatic Surgery, Casa di Cura Dott. Pederzoli, Via Monte Baldo 24, Peschiera del Garda Verona 37019, Italy

## Abstract

Appropriate surgical strategies for management of intraductal papillary mucinous neoplasms (IPMNs) of the pancreas are a matter of debate. Preoperative and intraoperative evaluation of malignant potential of IPMN and of patient's comorbidities is of paramount importance to balance potential complications of surgery with tumors' risk of being or becoming malignant; the decision about the extent of pancreatic resection and the eventual total pancreatectomy needs to be determined on individual basis. The analysis of frozen-section margin of pancreas during operation is mandatory. The goal should be the complete resection of IPMN reaching negative margin, although there is still no agreed definition of “negative margin.” Of note, the presence of deepithelization is often wrongly interpreted as absence of neoplasia. Management of resection margin status and stratification of surveillance of the remnant pancreas, based on characteristics of primary tumour, are of crucial importance in the management of IPMNs in order to decrease the risk of tumor recurrence after resection. Although risk of local and distant recurrence for invasive IPMNs is increased even in case of total pancreatectomy, also local recurrence after complete resection of noninvasive IPMNs is not negligible. Therefore, a long-term/life-time follow-up monitoring is of paramount importance to detect eventual recurrences.

## 1. Introduction

Described for the first time by Ohhashi and Murayama [1] in 1982 as a specific tumor-entity distinct from mucinous cystic neoplasms (MCNs) and ductal adenocarcinoma (PDA), intraductal papillary mucinous neoplasms (IPMNs) are increasingly being recognized. With the widespread use of cross-sectional imaging, IPMNs are nowadays frequently detected in asymptomatic individuals [2, 3]. Of note, in high-volume centers for pancreatic surgery, IPMNs represent one of the most common indications for pancreatectomy [4].

Histologically, IPMNs are mucin-producing neoplasms, arising from the main and/or secondary pancreatic ducts, with prominent intraductal growth and frequent papillary architecture [5]. The World Health Organization divided IPMNs into different entities, based on the involvement of pancreatic ductal system [5, 6]:* main-duct type *(MD-IPMN), when the tumor involves only the main pancreatic duct;* branch-duct type *(BD-IPMN), when the tumour involves only branch-ducts, with no macroscopic or microscopic involvement of the main pancreatic duct; and* mixed type*, when the neoplasia involves macroscopically and/or microscopically both the main pancreatic duct and its side branches. Because of their similar clinic-pathological characteristics and behaviour, mixed IPMNs are grouped with main-duct IPMNs in the patient's management [3]. On the basis of the degree of cytoarchitectural dysplasia [7, 8], IPMNs are associated with a spectrum of dysplastic changes of the epithelium, ranging from low-grade dysplasia (IPMN adenoma), intermediate-grade dysplasia (IPMN borderline), and high-grade dysplasia (IPMN with* carcinoma in situ)* that are considered to be noninvasive and IPMN with invasive carcinoma (invasive IPMN).

Furthermore, multifocal lesions can be found both in MD-, combined-, and BD-IPMNs [9]. BD-IPMNs present multifocal lesions in about half cases [3, 9]. Main pancreatic duct (MPD) can be entirely involved by the tumor that may extend along it or by synchronous skip lesions that are present in around 20% of the patients [10, 11]. In the light of the heterogeneity of this group of tumors and of their different risk of malignancy, it is important both preoperative and intraoperative evaluation of the malignant potential of the IPMN, in order to balance the risk of complications of pancreatic surgery with the IPMN risk of being or becoming malignant over time. The aim of this review is to analyze the factors influencing the extent of surgical resection and the implications of transection margin status during pancreatectomy for tumor recurrence and patient survival after surgery.

## 2. Management of IPMN

Guidelines based on experts' opinion and on the available evidence from the literature have been put forward by the International Association of Pancreatology in 2006 and revised in 2012 [6, 12] and by the European Study Group on Cystic Tumours of the Pancreas [13]. However, management of IPMNs is complex and several areas of uncertainty still remain in their treatment, both for the indications for surgical resection and for the surgical management of these patients.

### 2.1. Indications for Surgical Resection of Main Duct and Mixed IPMNs

In the light of the high frequency of malignancy and invasive carcinoma, even in patients without symptoms [14] or lacking radiological malignant parameters (enhanced solid component, MPD size of ≥ 10 mm) [12, 15], surgical resection is strongly recommended for all surgically fit patients with a clinic-radiologic diagnosis of MD- and combined-IPMNs [12], in order to achieve complete removal of the tumor with a negative margin. In consideration of tumor site and its extension along MPD, pancreaticoduodenectomy (PD), left pancreatectomy, or total pancreatectomy (TP) with splenectomy (LP) with lymph node dissection represent the treatment of choice [12].

### 2.2. Indications for Surgical Resection of Branch Duct IPMNs

Considering that the frequency of malignancy in resected BD-IPMN is around 25% and that these lesions mainly affect elderly patients with comorbidities increasing the surgical risk, a conservative management with continuous clinic-radiologic followup is recommended for the patients without risk factors for malignancy. Recent guidelines put forward by the International Association of Pancreatology (IAP) [12] identify (i) some “worrisome features” on imaging that include cyst of ≥ 3 cm, thickened enhanced cyst walls, MPD size of 5–9 mm, nonenhanced mural nodules, abrupt change in the MPD caliber with distal pancreatic atrophy, and lymphadenopathy and (ii) some “high-risk stigmata” for indication to resect (obstructive jaundice, enhanced solid component, MPD size of ≥10 mm).

In general, “high-risk stigmata” represent always a clear indication for surgical resection. On the other hand, BD-IPMNs without “high-risk stigmata” can undergo surveillance, with more strict follow-up timing for those patients with “worrisome features.” In a significant number of patients multiple BD-IPMNs can be detected (multifocal disease) [12, 16]. More than 50% of the patients with BD-IPMN present a multifocal disease with multiple BD-IPMNs along the gland [6]. There are no evidences of a higher rate of malignancy in multifocal BD-IPMNs [17]. In these cases, the approach follows the same criteria for the unifocal BD-IPMNs: when indication for surgical resection is present, standard segmental pancreatectomy can be performed if the lesions are confined to a single pancreatic region; otherwise, an extended resection up to a total pancreatectomy should be considered [12, 18, 19]. However, if the entire gland is involved, smaller lesions without malignancy-related features can be left behind and, instead of total pancreatectomy, a partial pancreatectomy should be performed [17].

### 2.3. Preoperative Planning of the Extent of Surgical Resection

In order to follow a correct oncologic approach, surgical treatment of an IPMN should be preceded by a careful study of the tumor topography and extent and of its possible signs of malignancy. Both computer tomography (CT) scan and magnetic resonance cholangiopancreatography (MRCP) are useful tools to assess the tumor and its relationship to nearby structures including peripancreatic vessels and to detect lymphadenopathy and/or distant metastases. Endoscopic ultrasound (EUS) is also a useful diagnostic tool to demonstrate thickness of the walls, mural nodules, and adjacent masses. EUS should be also combined with fine-needle aspiration in order to evaluate both cytology and pancreatic enzymes and tumoral markers (i.e., CEA and CA19-9). Moreover, ERCP can be used to demonstrate dilated pancreatic ducts and defects caused by mucin plugs or intraluminal neoplastic nodules; however, the slight risk of pancreatitis associated with this procedure must be considered.

Preoperative imaging is yet not always reliable to define the real entity of the tumor and its extension along the gland. So, at the operating theatre, the aim of surgical resection is the eradication of IPMN, and the intraoperative examination of the transection margin is of paramount importance to determine the necessity to proceed with a further pancreatic resection until the achievement of a resection margin free of epithelial atypia [20, 21], up to a possible total pancreatectomy [6, 9, 17, 20, 22, 23]. There is no agreement about the “right” extent of resection: some surgeons customarily resect all the gland involved by the tumor even if it results in total pancreatectomy and there is evidence of low-grade dysplasia, in order to avoid recurrence [21, 24]. On the other hand, an anatomic partial pancreatectomy, to preserve pancreatic parenchyma and to prevent metabolic consequences, is recommended by others [9, 25–27], thus stopping the resection once no high-grade dysplasia is present.

### 2.4. Parenchyma-Sparing Pancreatectomy

Parenchyma-sparing resections of the pancreas include enucleation, middle pancreatectomy (MP), and middle-preserving pancreatectomy [28–31]. In order to decrease the risk of development of exocrine/endocrine insufficiency, some patients may benefit from these “atypical” resections [31, 32].

#### 2.4.1. Enucleation

It has been proposed for “low-risk” BD-IPMN [31]. However, the great majority of low-risk BD-IPMNs can be safely managed nonoperatively, while in “high-risk” BD-IPMNs a formal pancreatectomy should be performed. Moreover, in patients with a suspected BD-IPMN at preoperative imaging undergoing enucleation, a proper histological examination of the connection with the MPD cannot be made, and a microscopic involvement of the MPD (combined IPMN) cannot be excluded. In the cases reported in the literature, no patient treated with enucleation developed tumor recurrence, but again, most of these patients had benign BD-IPMNs that could be also managed nonoperatively. On the other hand, a higher fistula rate has been reported compared to standard resections [33].

#### 2.4.2. Middle Pancreatectomy (MP)

It can be performed for BD-IPMNs in the neck or proximal body of the pancreas without malignancy-related features but with an indication for surgical resection. In this case, after an accurate preoperative study of the lesion to exclude clinical and radiological signs of malignancy, intraoperative histological examination of the two resection margins is mandatory; if a frozen-section examination is positive for malignant disease, the operation must be converted in a standard pancreatectomy. Perioperative morbidity after MP is higher than that for standard pancreatectomy [34], but, considering the preservation of pancreatic function, MP is an effective alternative to formal pancreatic resection [35]. MP has been proposed also for MD- or combined-IPMN involving exclusively the neck of the pancreas. Again, the intraoperative examination of both the transection margins must be performed. However, the recurrence rate after MP for MD-IPMNs is significant (33%) [35], limiting the role of MP for these tumors.

#### 2.4.3. Middle-Preserving Pancreatectomy

It can be an effective alternative to total pancreatectomy, decreasing the risk of pancreatic insufficiency, when the lesions involve all pancreas except the body, as it could happen in multifocal IPMNs. After an intraoperative ultrasound, pancreaticoduodenectomy and distal pancreatectomy with splenectomy are performed leaving the pancreatic body: the two section margins are sent for frozen-section examination [31]. If tumor involvement is present, total pancreatectomy should be performed.

## 3. Transection Margin Status

### 3.1. The Definition of “Positive” Margin

There is no consensus about the definition of “positive” resection margin in case of pancreatectomy for IPMNs, and the lack of a clear definition implies a great heterogeneity among different studies.

Some authors classify surgical margins in IPMN as “negative” in case of presence of normal epithelium or mucinous hyperplasia without dysplasia in the main duct and as “positive” in case of adenoma, borderline neoplasm, or carcinoma [17].

Other authors instead follow another classification presenting negative resection margin (with normal columnar epithelium or denuded), mucinous hyperplasia (pancreatic intraepithelial neoplasia Pan-IN 1A or 1B), or positive resection margin (dysplasia Pan-IN 2 or carcinoma Pan-IN 3) [9, 36].

Considering the degree of dysplasia, in some studies, the surgical margin is reported as negative if it presents normal epithelium or IPMN adenoma and as positive in case of moderate or severe dysplasia (borderline IPMN or carcinoma* in situ* IPMN) [37].

Otherwise, a lesion can be considered “significant,” thus requiring additional resection, in presence of at least IPMN adenoma on the main duct or at least borderline IPMN on branch ducts [38].

One of the factors most clearly associated with recurrence is the presence of deepithelialization (denudation) at the resection margin that is so wrongly interpreted as an absence of neoplasia [20, 38]. The presence of denudation should routinely lead to an extension of surgical resection [38], since it is associated with an increased rate of recurrence [39].

During frozen section analysis of the resection margin, there is also the possibility to find incidental PanIN lesions that are not always simple to distinguish from IPMN extending into small ducts. They are both intraductal proliferations of mucin-producing cells that may possess various degrees of atypia and have the potential to progress to adenocarcinoma [7]. PanINs are usually incidental microscopic findings associated with smaller ducts [7, 40]. The authors who performed additional resection only for high-grade dysplasia or invasive carcinoma classify PanIN eventuality as “no significant dysplasia” at the margin [41].

### 3.2. Intraoperative Implications of Transection Margin Status

Intraoperative analysis of the transection margin during pancreatectomy for an IPMN is important but also controversial because of its implications. In case of an IPMN involving, at the preoperative imaging, only a segment of the pancreas, the chance that the radiological imaging could underestimate the real extent of ductal involvement makes mandatory an intraoperative frozen-section (FS) histological examination to define the appropriate cut line [20, 38, 42–44]. When the intent of the pancreatectomy is a curative resection, many authors emphasize the importance of obtaining a tumor-free surgical margin by FS analysis [21, 25, 45, 46].

IAP guidelines suggest that when adenoma (low-grade dysplasia) is present at the resection margin, no further resection is required because the risk of progression to cancer or local recurrence is minimal; instead, moderate- or high-grade dysplasia as well as invasive carcinoma at the FS requires an additional resection, up to a total pancreatectomy [12].

However, in the light of the different definitions of “positive” margins, in the literature the optimal surgical strategy remains controversial.

In the study of Couvelard et al. [38], the result of the FS analysis implies an extent of the resection in 30% of the patients, allowing an adequate resection in 97% of the cases; similar percentage of additional resection is presented by Salvia et al. (21%) [14].

The percentage of concordance between FS and definitive examination of the margins is different in the various studies but tends to be high: all the intraoperative diagnoses have been confirmed at the final pathologic analysis in the study of Salvia et al. [14]; moreover, high accuracy rate has been reported by Fujii et al. (99%) [47], Raut et al. (97%) [48], and Couvelard et al. (94%) [38]. Nevertheless, lower percentages of concordance have been reported in the series of White et al. (67%, with a positive predictive value of frozen section of 50% and a negative predictive value of 74%) [22] and in the study of Frankel et al. (57%, with a positive predictive value of 41.2% and a negative predictive value of 66.7%) [49]. The case of misdiagnosis presented by Raut et al. [48] concerns a pancreaticoduodenectomy for noninvasive IPMN: while the FS margin was interpreted as negative, the final pathology report revealed the presence of a microscopic focus of noninvasive IPMN. The patient did not undergo reresection and did not develop recurrent disease. Regarding the conflicting results in the study of Couvelard et al. [38], even if 9 cases of “underestimation” and 3 cases of “overestimation” by FS are reported, only 4 patients (3%) have had inadequate extent of the pancreatic resection, excessive in one case and insufficient in 3 cases, consisting in normal epithelium versus IPMN adenoma or borderline IPMN or noninvasive carcinoma in main duct at the definitive examination. In the paper further resections for these patients are not reported. As a general recommendation, surgical reexploration and pancreatic resection should be considered when malignancy is found at final pathology on the resection margin, given the high risk of recurrence in this setting. The possibility of performing a total pancreatectomy must be carefully discussed with the patient. If the patient refuses the reoperation, a very strict radiological followup is mandatory on a 3-4-month schedule. When benign IPMN is found at final pathology on the resection margin, long-term clinic-radiological followup is preferable to immediate reresection.

When we look at the data on total pancreatectomy, the procedure has been performed heterogeneously, ranging from 2.7% [22] to 23% [9].

The analysis of FS margin is indicated in all IPMNs, but its implications on MD-IPMN and BD-IPMN are different. In BD-IPMNs, it is important to analyze the resection margin in order to exclude the presence of the tumor, but extension of the surgical resection is uncommon [38]. In MD- and combined-IPMNs, instead, frozen-section margin analysis is of paramount importance because dilatation of the MPD and neoplasia of the duct lining are not always correlated. In fact, dilatation of MPD can be due only to pancreatitis and obstruction by mucus upstream or downstream from the tumor [38]. The task of the pathologist is to deem the question with the microscopic analysis of the margin.

Moreover, the risk of a positive margin seems to be correlated with the degree of IPMN dysplasia, being significantly higher in patients with moderate- or high-grade dysplasia (50%) than in patients with low-grade dysplasia (22%) [49].

### 3.3. Pancreatoscopy

Since IPMN can arise in multiple sites within the pancreas and preoperative imaging can be inadequate in detecting microscopic spread of cancerous lesions or skip lesions [37], it is fundamental to evaluate if FS margin analysis is the only factor to be considered in determining the extent of the resection.

The knowledge of skip lesions and the development of recurrence in patients with noninvasive IPMN and negative surgical margin suggest that the real extension of the IPMN involvement of the pancreatic gland can be difficult to predict.

Hara et al. [50] recommend the combination of peroral pancreatoscopy and intraductal ultrasonography for an improved differential diagnosis between malignant and benign IPMNs [9].

A further help in planning extent of the resection can be provided by pancreatoscopy with narrow band imaging (NBI) that is done using flexible pancreatoscope through the cut end of the duct at the surgical margin after partial pancreatectomy. Kaneko et al. [11] have reported the incidence of multicentric lesions as high as 20.8%, with high rates of sensitivity and specificity for the procedure. Intraoperative pancreatoscopy allows an accurate examination of the entire duct and NBI facilitates in better identification of the vascular pattern of the lesion. The intraoperative pancreatoscopy with NBI access to main duct seems to be more accurate than peroral pancreatoscopy [27].

In order to detect skip lesions and hence the real intraductal tumor extension, an intraoperative 2- or 3-segmental cytology of the pancreatic juice, in addition to frozen-section analysis, can be performed [51–53]. A single-lumen catheter is inserted across the cut surface in the main pancreatic duct of the cranial pancreas and a triple-lumen catheter is inserted into the caudal pancreas to obtain the pancreatic juice separately from each portion of the pancreatic head, body, and tail. After cytological analysis, segments with positive cytology should be additionally resected. In the study of Eguchi et al. [37], all patients with positive cytology and negative surgical margins had skip lesions in further resected specimens. After histological and cytological examinations, 42% of the patients required additional resection. No patient developed a recurrence in the remnant pancreas. However, although these data are promising, they need to be confirmed in larger cohorts of patients.

## 4. Recurrence and Survival after Surgical Resection

Tumor recurrence after pancreatic resection for IPMN can be classified as local, regional, or distant (metastatic). Local recurrence is defined as the presence of an IPMN in the pancreatic remnant after partial pancreatectomy [9]. Recurrence after resection of noninvasive IPMN may occur because of (1) a residual dysplastic tissue at the surgical margin, (2) a multicentric tumor with synchronous skip lesions in the remnant pancreas undetected during initial operation, or (3) metachronous lesions that have developed in the remnant pancreas as a result of a neoplastic tendency to involve the entire gland (field defect) [9, 23, 42, 54].

Chari et al. [9] analyzed a group of 133 patients resected for noninvasive IPMN (73 patients) and invasive IPMN (40 patients) and proposed a correlation between recurrence/survival after surgical resection and the histology of the tumor. Eight percent of noninvasive IPMN showed recurrence after partial pancreatectomy, after a median followup of 37 months and none of the 13 patients who underwent total pancreatectomy had extrapancreatic recurrence. In invasive IPMNs, recurrences were similar both after partial pancreatectomy and total pancreatectomy (67% and 62%, resp.), and 91% of them occurred within 3 years from surgery. Five-year survival was higher in noninvasive (84.5%) than in invasive IPMNs (36%). 26% of the patients showed a recurrence in the pancreatic bed or in the remnant pancreas, whereas 74% had either a distant metastatic recurrence or both local and distant metastatic recurrence. The most common metastatic site was the liver (65%). In case of invasive IPMN, the presence of dysplasia at the margin was the only predictor of recurrence.

D'Angelica et al. [55] presented a series of 63 patients with IPMN surgically managed. Of these, 51% had resection margins involved with atypia or carcinoma* in situ*; however, the presence of mild- or borderline dysplasia or carcinoma* in situ* at the resection margin was not associated with a poor outcome. 23% of the resected patients developed a recurrent disease, with half of these in the first 2 years. The median time from surgery to recurrence was 20 months. The rate of disease specific outcomes did not differ among patients with and without positive margins. Disease-specific 5-year survival was 75%. Significant predictors of poor outcome included elevated serum total bilirubin, presence of invasive carcinoma with its extent and type (tubular versus colloid), lymph node metastases, and vascular and perineural invasion. These factors were all significantly associated with the recurrence of tumor, unlike the margin status that was not associated with disease recurrence. Then, early oncologic outcome was determined by the pathologic characteristics of the primary tumor and not by the resection margin status.

Falconi et al. [20] showed a local recurrence in 8% of 51 patients with IPMN treated by pancreatic resection. Mild to moderate dysplasia was present at the frozen-section margin in 20 specimens (41%) and carcinoma in one.

All the patients with recurrence underwent a second resection. The 3-year survival rate for benign IPMNs was 94% and 69% for malignant ones. In this paper, the importance of the presence of deepithelialization of the resection margin was highlighted; local recurrence in patients with eroded epithelium at the surgical margin must lead to considering deepithelialization of the margin as a “positive resection margin.”

Frankel et al. [49], in a study with 192 patients undergoing resection of noninvasive IPMN, showed a recurrence of 21% at a median followup of 46 months. Ductal dysplasia at the final surgical margin was defined by the presence of IPMN or PanIN, regardless of the degree of dysplasia. 31% of the patients with margin dysplasia recurred, whereas, among patients without dysplasia, 13% presented recurrent disease. However, this was not associated with poor survival. Of note, tumor recurrence was not found at the level of the surgical margin but in the remnant pancreas, far from transection line. Dysplasia at the resection margin was associated with recurrence in the remnant gland, but not at the resection margin. According to the authors, this indicates that, albeit a positive margin is associated with recurrence, it is more likely a marker of diffuse ductal instability and not a local oncologic failure.

Fujii et al. [47] considered 103 cases of noninvasive IPMNs including* carcinoma in situ* (CIS). Recurrences were observed in 4.9% of the patients with benign IPMN and in 22.7% of the patients with CIS; none recurred at the resection margin, 9 recurred in the remnant pancreas, and 1 at the peritoneal surface, probably for the preoperative EUS-guided fine-needle aspiration biopsy. The presence of adenoma at the resection margin seems to have no influence on the outcome, because recurrence was diagnosed in 7.8% of adenoma-negative patients and in 10.7% of adenoma-positive patients and overall survival and recurrence-free survival were similar between the two groups.

Salvia et al. [14] analyzed 140 patients with MD-IPMN (with or without side branch involvement). The rate of recurrence after resection in the remnant pancreas was 7%; only one patient did not have invasive cancer as primary tumor. Patients with noninvasive IPMN had a 5- and 10-year cancer-specific survival of 100%, whereas for patients with invasive carcinoma 5- and 10-year survival was 60% and 50%, respectively. Among the 32 patients with a positive or indeterminate resection margin, 4 (8%) developed a late recurrence in the remaining pancreas.

Schnelldorfer et al. [56] analyzed 208 patients with IPMN; 58% of the invasive IPMNs recurred, whereas 10% of noninvasive IPMNs recurred after partial pancreatectomy and 0% after total pancreatectomy. Five-year survival in patients with noninvasive IPMN was 94%; instead, five-year survival in patients with invasive IPMN did not differ much from 5-year survival of a matched cohort with ductal adenocarcinoma (31% versus 24%).

In case of negative margin of resection, the median survival was 119 months and the 5-year survival rate was 77% and those were greater but not statistically significantly different from those of patients with “benign” positive margin (62 months and 52%). Otherwise, patients with malignant positive margin had the worse survival rate (median survival 11 months and 5-year survival rate 0%).

Sohn et al. [54], considering 136 pancreatic resections for patients with IPMNs, found an overall 5-year survival for patients with IPMN without invasive cancer of 77% and of 43% in patients with an invasive component.

There were no differences in survival between patients with different dysplasia in the primary tumor (adenoma, borderline neoplasms, and CIS) and neither comparing BD-IPMNs, MD-IPMNs, and combined variants.

White et al. [22], in a series of 78 patients resected for noninvasive IPMN, found that there was no significant difference in local recurrence rates between BD-IPMNs (7.9%) and MD-IPMNs (7.5%). Local recurrence was described in 7.7% of the patients at a median followup of 40 months, with a median interval of 22 months from the resection. Only 2% of the patients with negative margins recurred, whereas 17% with positive margins presented recurrence, and thus also the majority of patients with positive margin of resection did not develop local recurrence; anyhow, local recurrence free survival was significantly higher for patients with negative margin than for patients with positive margin. Regarding PanIN-1 or -2, no patients with these lesions at the resection margin presented recurrence. In case of borderline or* carcinoma in situ* IPMN as primary tumor, the percentage of positive margin for IPMN was higher than that in case of patients with adenoma (42% versus 9%, resp.). The estimated 5-year local recurrence-free survival for all patients resected was 87%.

Tables 1 and 2 summarize the main data of the abovementioned studies.

Data in Tables 1 and 2 show a difference in the behavior of invasive and noninvasive resected IPMNs. In case of noninvasive tumors, even if the percentage of positive margin is significantly different in the various studies, the percentage of recurrence rate and 5-year survival can be considered quite similar, that is, uncommon recurrence and good prognosis, regardless of the positivity of resection margin. Otherwise, when the resected tumors are invasive, data are significantly different in the studies, suggesting that other factors, in addiction to resection, determine the prognosis.

## 5. Followup

IPMN is often a slow-growing tumor, so recurrences, if any, can occur late after resection and might be underestimated after short-term followup [9, 57–59]. Therefore, long-term or, probably, life-time followup with surveillance imaging is required [9, 56]. However, there are no clear indications regarding the frequency, duration, or methods of postoperative surveillance in patients with resected noninvasive IPMNs [60, 61].

Patients with noninvasive IPMN after partial pancreatectomy with negative margins must be informed about the risk of recurrence and, in case of appearance of symptoms as abdominal pain, pancreatitis, jaundice, new-onset steatorrhea, or weight loss, they should be investigated promptly with a radiologic study (CT or MRI) [9].

The frequency of followup should be adjusted based on the risk of recurrence. Patients with IPM-adenoma, negative resection margin, and normal remnant pancreas on postoperative imaging can follow a yearly or biannual surveillance. For patients with IPM-borderline or CIS and a positive resection margin or indeterminate cystic lesions in the remnant pancreas, the surveillance should be more frequent for the first 2 to 3 years (twice a year), when most of recurrences usually occur [12]. In any case, indefinite radiographic surveillance must be recommended, because recurrences might occur even more than 5 years after resection [9, 14, 22, 54].

For patients with invasive IPMN, the indicated followup should be identical to that for ductal adenocarcinoma with surveillance every six months [12].

It has been suggested that patients with IPMN present an increased risk of developing additional pancreatic and extrapancreatic malignancies [62–65], which may occur before, after, or concurrent with the diagnosis of IPMN. PDAC is detectable in 2–10% of patients with BD-IPMN, in separate regions from the IPMN [66, 67]. The most common extrapancreatic malignancies are gastric and colorectal cancers [41, 68–70]; therefore, surveillance for these secondary malignancies must be maintained in these patients [37, 70].

## 6. Conclusions

All these data confirm that resection is the treatment of choice for MD- and combined-IPMN and for BD-IPMN with high-risk stigmata. The analysis of frozen-section margin of pancreas during the operation is mandatory, and it plays a role in the proper management of MD- and combined-IPMNs. In case of invasive cancer or intermediate/high-grade dysplasia, an additional resection, up to eventual total pancreatectomy, should be performed.

The percentage of concordance between FS and definitive examination of the margins tends to be high in the various studies; anyway, in the literature, there are no guidelines for the event of a conflicting result between FS and definitive examination of the resection margin. At any rate, as general recommendation, surgical reexploration and pancreatic resection should be considered when malignancy is found at final pathology on the resection margin for the high risk of recurrence in this setting. The possibility of a total pancreatectomy must be carefully discussed with the patient. If the patient refuses the reoperation, a strict radiological followup every 3-4 months is recommended. When benign IPMN is found at final pathology on the resection margin, long-term clinical and radiological followup is preferable to immediate reoperation.

Recurrence after complete resection of noninvasive IPMNs is uncommon but not negligible, regardless of the degree of epithelial dysplasia in the neoplasm. There is an increased risk of local, regional, or distant metastatic recurrence for invasive IPMNs even in the setting of “curative” resection, usually within the first 3 years of resection. Liver is the most common site of metastatic recurrence, and total pancreatectomy does not prevent the recurrence of cancer. Furthermore, in the light of the possible severe metabolic consequences, prophylactic total pancreatectomy is not recommended in patients with apparently limited disease and negative margins. Total pancreatectomy should be strongly considered in young, fit patients with high-grade dysplasia (including at least Pan-IN 3) or invasive cancer at the resection margin. It is evident that the decision about the extent of pancreatic resection and the eventual total pancreatectomy needs to be determined on individual basis, considering the preoperative patient's status and the presence of comorbidities, symptoms, or an already existing insulin-dependent diabetes.

Of note, the presence of deepithelization is often considered as absence of neoplasia; however, the association of “denuded” epithelium at the resection margin with tumor recurrence suggests that it is wrongly interpreted as negative margin. In these cases, an extension of surgical resection is mandatory.

A critical point in the management of patients with IPMNs is the postoperative surveillance. Recurrence can be due to a multifocal disease, with a synchronous, undetected, IPMN present within the remnant pancreas or because of the development of a metachronous IPMN as a result of a widespread neoplastic field defect in the pancreatic ductal epithelium. Therefore, follow-up monitoring is of paramount importance to detect recurrence. Current guidelines recommend stratification of surveillance of the remnant pancreas based on characteristics of primary tumor. Patients with a high risk of recurrence should be assessed every 3–9 months with cross-sectional imaging, whereas low-risk patients may be screened annually/biannually. Since recurrences can occur even 10 years or more after resection, a long term/life-time followup after resection of IPMN is mandatory.

## Figures and Tables

**Table 1:** Characteristics of noninvasive resected IPMNs.

Author	Total *n* (%) noninvasive IPMN	Positive margin, %	Recurrence rate, %	5-year survival, %	Median follow-up, months
Chari et al., 2002 [9]	73 (65)	3.3	8	84.5	36
D'Angelica et al., 2004 [55]	32 (52)	51.6 (noninv + inv)	4.8	91	32
Falconi et al., 2001 [20]	32 (63)	36.7	8 (noninv + inv)	94 (3-year surv.)	15 (mean)
Frankel et al., 2013 [49]	192 (100)	45	21	32.3	46
Fujii et al., 2010 [47]	103 (72)	27.2	9.7	—	41
Salvia et al., 2004 [14]	72 (51)	22.2	1.4	100	31
Schnelldorfer et al., 2008 [56]	145 (70)	2.8	10	94	—
Sohn et al., 2004 [54]	84 (62)	24	8.3	77	24 (mean)
White et al., 2007 [22]	78 (100)	29.5	7.7	87	40

**Table 2:** Characteristics of invasive resected IPMNs.

Author	Total *n* (%) invasive IPMN	Positive margin, %	Recurrence rate, %	5-year Survival,%	Median follow-up, months
Chari et al., 2002 [9]	40 (35)	26	65	36	42
D'Angelica et al., 2004 [55]	30 (48)	51.6 (noninv + inv)	14.5	58	32
Falconi et al., 2001 [20]	19 (37)	79	8 (noninv + inv)	69 (3-year surv.)	15 (mean)
Salvia et al., 2004 [14]	58 (41)	27.6	12.1	60	31
Schnelldorfer et al., 2008 [56]	63 (30)	28.6	58	31	—
Sohn et al., 2004 [54]	52 (38)	38.5	—	43	24 (mean)
